# Empirical assessment of analysis workflows for differential expression analysis of human samples using RNA-Seq

**DOI:** 10.1186/s12859-016-1457-z

**Published:** 2017-01-17

**Authors:** Claire R. Williams, Alyssa Baccarella, Jay Z. Parrish, Charles C. Kim

**Affiliations:** 1 Department of Biology, University of Washington, Seattle, WA 98195 USA; 2 Division of Experimental Medicine, Department of Medicine, University of California, San Francisco, CA 94143 USA; 3 Present address: Verily, South San Francisco, CA 94080 USA

**Keywords:** Monocytes, Classical, Nonclassical, RNA-Seq, Gene expression analysis

## Abstract

**Background:**

RNA-Seq has supplanted microarrays as the preferred method of transcriptome-wide identification of differentially expressed genes. However, RNA-Seq analysis is still rapidly evolving, with a large number of tools available for each of the three major processing steps: read alignment, expression modeling, and identification of differentially expressed genes. Although some studies have benchmarked these tools against gold standard gene expression sets, few have evaluated their performance in concert with one another. Additionally, there is a general lack of testing of such tools on real-world, physiologically relevant datasets, which often possess qualities not reflected in tightly controlled reference RNA samples or synthetic datasets.

**Results:**

Here, we evaluate 219 combinatorial implementations of the most commonly used analysis tools for their impact on differential gene expression analysis by RNA-Seq. A test dataset was generated using highly purified human classical and nonclassical monocyte subsets from a clinical cohort, allowing us to evaluate the performance of 495 unique workflows, when accounting for differences in expression units and gene- versus transcript-level estimation. We find that the choice of methodologies leads to wide variation in the number of genes called significant, as well as in performance as gauged by precision and recall, calculated by comparing our RNA-Seq results to those from four previously published microarray and BeadChip analyses of the same cell populations. The method of differential gene expression identification exhibited the strongest impact on performance, with smaller impacts from the choice of read aligner and expression modeler. Many workflows were found to exhibit similar overall performance, but with differences in their calibration, with some biased toward higher precision and others toward higher recall.

**Conclusions:**

There is significant heterogeneity in the performance of RNA-Seq workflows to identify differentially expressed genes. Among the higher performing workflows, different workflows exhibit a precision/recall tradeoff, and the ultimate choice of workflow should take into consideration how the results will be used in subsequent applications. Our analyses highlight the performance characteristics of these workflows, and the data generated in this study could also serve as a useful resource for future development of software for RNA-Seq analysis.

**Electronic supplementary material:**

The online version of this article (doi:10.1186/s12859-016-1457-z) contains supplementary material, which is available to authorized users.

## Background

RNA sequencing (RNA-Seq) has become the preferred technique for transcriptome-wide analysis of gene expression. However, estimating expression from short sequence reads poses unique problems such as accurate read alignment in the presence of sequencing errors, measurement bias depending on library preparation methodology, and complexity in estimating the expression of distinct mRNA transcripts with shared exons. As a result, RNA-Seq analysis is still rapidly evolving, with a wide number of tools available for each of the major processing steps, and many combinations in which these tools are commonly implemented. As such, the optimal workflow for a given application remains a subject of intensive investigation.

The most typical application of RNA-Seq is the identification of differentially expressed genes. In such an analysis, two or more conditions are compared to identify changing gene expression signatures, from which functional changes or markers of a given cellular state are inferred. The three major steps of differential expression analysis by RNA-Seq are alignment of reads to an annotated genome (or less commonly, *ab initio* reconstruction of a transcriptome annotation [1, 2]), expression modeling to obtain gene-level and/or transcript-level expression estimates, and statistical analysis to identify differentially expressed genes or transcripts between comparison groups [3–8]. Various studies have evaluated the performance of the available tools at each isolated step of this workflow [9–18]; however, only a handful of studies have evaluated the performance of these approaches in concert with one another [3, 19, 20]. This is important since upstream processing could have substantial effects on downstream steps and outcomes [21]. In addition, performance has largely been evaluated using controlled datasets, such as those from highly purified reference RNA samples, cell lines, or reads synthetically derived *in silico*. These datasets often exhibit extreme differences in gene expression between sample groups that are unrepresentative of more typical experimental designs in which the control and test samples are more closely related to one another. In addition, such datasets do not possess the inter-sample variability in sequencing depth and quality that often occurs in many real-world settings. This is particularly true when clinical samples are involved, for which there is typically more variability in the initial sample quality, and for which analysis must also tolerate genetic variation. Thus, although such comparisons are valuable for initial benchmarking of a given algorithmic approach and its implementation, the ultimate evaluation of any given tool must take into consideration the samples to which it will be applied and the workflow context in which it will be employed.

One of the barriers to validating analysis workflows is a paucity of real-world RNA-Seq samples for which reference datasets are available for comparison. Here, we describe an RNA-Seq dataset generated from human classical and nonclassical monocyte subsets isolated to high purity. Differential gene expression analysis between these subsets has been analyzed in multiple transcriptome-wide microarray and BeadChip studies [22–25], providing us with gene sets that have been validated by multiple independent laboratories using multiple gene expression analysis platforms. Therefore, these gene sets provide a reference estimate of biological ‘truth’. Using the sequence reads from our monocyte subset dataset, we evaluated commonly used differential expression workflows for their performance, as assessed by their agreement with these references. We find that different RNA-Seq analysis workflows differ widely in their performance, as assessed by recall, or the proportion of reference-identified genes that were also identified by the given workflow, and precision, or the proportion of genes identified by the workflow that were also identified by the reference. Many workflows perform equally well, but are calibrated differently with respect to favoring higher recall or precision, with an inverse relationship between these parameters. Based on our observations, we recommend that the selection of a given approach be guided by the tolerance of downstream applications for type I and type II errors. Used in conjunction with the previous microarray and BeadChip studies, these RNA-Seq data provide a real-world test set for guiding the development of improved software and workflows.

## Methods

### Samples

Blood was collected from Ugandan children as part of the *Program for Resistance, Immunology, Surveillance & Modeling of Malaria in Uganda* study using previously described methods [26]. Peripheral blood mononuclear cells (PBMCs) from a total of 18 individuals were isolated on Ficoll gradients, counted, and immediately cryopreserved and stored long-term in liquid nitrogen. Samples were thawed in the presence of DNase and immediately stained in FACS buffer with antibodies specific for the following targets: CD7 (clone 4H9), HLA-DR (clone L243), CD16 (clone CB16), CD14 (clone 61D3), CD19 (clone HIB19) from eBioscience; and CD177 (clone MEM-166) from Biolegend. For flow cytometry, classical monocytes were identified as CD177^−^CD7^−^CD19^−^HLA^−^DR^+^CD14^hi^CD16^−^; nonclassical monocytes were identified as CD177^−^CD7^−^CD19^−^HLA^−^DR^+^CD14^lo^CD16^+^. Both monocyte subsets were isolated to high purity using two consecutive rounds of sorting on a FACSAria, using an event rate no higher than 5,000 events/s and sorting directly into an RNA preservative buffer on the second sort. A total of 67 – 3149 cells were sorted per sample. Each sample represents a single individual, and both nonclassical and classical subsets were sorted from each individual. Sorted cells were immediately snap frozen on dry ice and stored in a −80 °C freezer until the time of RNA isolation.

### RNA sequencing

Cryopreserved sorted cells were thawed, and RNA was isolated using an RNAqueous Micro kit (ThermoFisher, Waltham, MA) following manufacturer recommendations with the following modifications: lysis buffer/cell aliquots were initially mixed with 180 μL of 200 proof RNase-free ethanol; the flowthrough was reloaded onto the column to capture additional material with a second binding step; and the purified RNA was eluted twice with 6 μL 55 °C RNase-free water following a 2 min incubation. Isolated total RNA was vacuum concentrated to 1 μL and converted to pre-amplified cDNA libraries using template-switching reverse transcription [27, 28] as implemented in the SMARTer Ultra-low input kit (Clontech, Mountain View, CA). Two samples failed to yield cDNA and were thus excluded from further processing. Fragmentation was performed enzymatically using a Nextera XT DNA kit (Illumina, San Diego, CA), and barcoded samples were multiplexed, pooled, and purified using Agencourt AMPure XP beads (Beckman Coulter, Brea, CA). Libraries were quality-controlled for size distribution and yield using a Bioanalyzer 2100 with high sensitivity dsDNA assay (Agilent Technologies, Santa Clara, CA), and sequenced as 51 bp single-end reads on 4 lanes of a HiSeq 2500 (Illumina) running in high-output mode at the UCSF Center for Advanced Technology (San Francisco, CA). Reads were demultiplexed with CASAVA (Illumina), and read quality assessed using FastQC [29].

### Read alignment, expression modeling, and differential expression identification

Reads were aligned to release GRCh37 of the human genome. Reads were aligned with Bowtie2, HISAT2, Kallisto, Salmon, Sailfish, SeqMap, STAR and TopHat2 [30–38]. Gene and transcript expression was estimated with BitSeq, cufflinks, htseq, IsoEM, Kallisto, RSEM, rSeq, Sailfish, Salmon, STAR, Stringtie and eXpress [32–35, 37, 39–45]. The IsoEM code was modified to increase the maximum available memory. Expression matrices for differential expression input were generated using custom scripts as well as the prepDE.py script provided at the Stringtie website. Differentially expressed genes or transcripts were identified with Ballgown, baySeq, BitSeq, cuffdiff, DESeq2, EBseq, edgeR exact test, limma coupled with vst or voom transformation, NBPseq, NOISeqBIO, SAMseq and Sleuth [33, 39, 40, 46–54]. Of these, all but Ballgown, BitSeq, NBPSeq, SAMSeq, and Sleuth used intrinsic filtering or recommended extrinsic filtering of genes or transcripts prior to testing. For Sailfish and Salmon, outputs were converted to a Sleuth-ready format using wasabi [55]. For Kallisto, Sailfish, Salmon, and BitSeq, transcript-level values were condensed to gene-level values using tximport prior to evaluating gene-level differential expression [56]. For all differential expression analyses performed at the transcript-level, significant transcripts were converted to the corresponding gene for performance evaluation, such that if a single transcript was called as differentially expressed, the corresponding gene was also called differentially expressed. We note that because of this unavoidable difference between gene-level and transcript-level comparisons, quantitative comparisons of recall and/or precision between a gene-level and a transcript-level workflow should be avoided. Rather, we recommend evaluating the relative performance of a given workflow as compared with other workflows with matched gene-level or transcript-level estimation. When possible, differential expression was assessed using multiple expression units (counts, FPKM, TPM) and performance metrics are reported separately for each unit. In general, all software was run with default parameters; specific runtime parameters are listed in Additional file 1, along with software versions, and scripts for running all code are available at https://github.com/cckim47/kimlab/tree/master/rnaseq. Further information about implementation is available upon request. All software was run at a detection level of alpha of 0.05, FDR of 0.05, or PPLR in the most extreme 0.05. Abbreviations used throughout the figures are a six-letter code represented as AaBbCc, where Aa denotes the read aligner (RA), Bb denotes the expression modeler (EM), and Cc denotes the differential expression (DE) analysis tool. All tools and codes are shown in Table 1.

**Table 1:** Analysis tools used in this study

Read aligner	RA code	Expression modeler	EM code	Differential expression	DE code
Bowtie2	Bw	BitSeq	Bs	Ballgown	Bl
HISAT2	Hs	cufflinks	Cu	BitSeq	Bs
Kallisto	Ka	htseq	Ht	baySeq	By
Salmon-FMD	Sf	IsoEM	Ie	cuffdiff	Cd
Sailfish	Sl	kallisto	Ka	DESeq2	De
SeqMap	Sm	RSEM	Rm	EBseq	Eb
Salmon-Quasi	Sq	rSeq	Rs	edgeR	Er
STAR	Sr	Sailfish	Sl	limma + voom	Lo
TopHat2	Th	Salmon	Sn	limma + vst	Lv
		STAR	Sr	NBPseq	Nb
		Stringtie	St	NOISeqBIO	No
		eXpress	Xs	SAMseq	Sa
				Sleuth	Su

Abbreviations specified in the table are used throughout the figures. Additional details are available in Additional file 1

### Preparation of reference datasets

Reference datasets were prepared from four published studies conducted on microarray or BeadChip platforms (GSE25913, GSE18565, GSE35457, GSE34515) [22–25]. An additional reference set (GSE16836 [57]) was considered, but excluded due to inter-sample variation precluding identification of differentially expressed genes. Significant differentially expressed genes between classical and nonclassical monocytes were identified for each dataset. In brief, series matrix files were downloaded from the NCBI Gene Expression Omnibus, log_2_ transformed if necessary, full-quantile normalized [50], and analyzed for statistically significant gene expression between classical and nonclassical monocytes. To reduce bias introduced by a single statistical method, we employed two approaches: Significance Analysis of Microarrays (SAM) [58] with a false discovery rate of 0.05, and limma [59, 60], with a BH-adjusted p-value of 0.05. Performance of the workflows against both SAM and limma were compared to one another and found to exhibit good reproducibility regardless of the statistical method used to generate the data (Additional file 2 and Additional file 3); as such, we chose to use the genes at the intersection of the two methods for our final reference gene sets.

### Quantification of recall and precision

Because absolute recall and precision values are influenced by the repertoire of analytes that can be measured by a given platform, we first filtered each reference and RNA-Seq gene set to include only features measurable both by RNA-Seq (i.e., present in the GRCh37 genome release) and by the microarray (i.e., a probe targeting the feature was present on the microarray platform) within a given comparison. All gene set counts are reported based on these filtered numbers, as are all estimates of recall and precision. Recall was calculated as the number of significant genes in the intersection of the test RNA-Seq dataset with the reference dataset, divided by the number of genes identified as significant in the reference dataset. Precision was calculated as the number of significant genes in the intersection of the test RNA-Seq dataset with the reference dataset, divided by the number of genes identified as significant in the test RNA-Seq dataset.

## Results and discussion

### Generation of a real-world RNA-Seq dataset for benchmarking

**Fig. 1:**
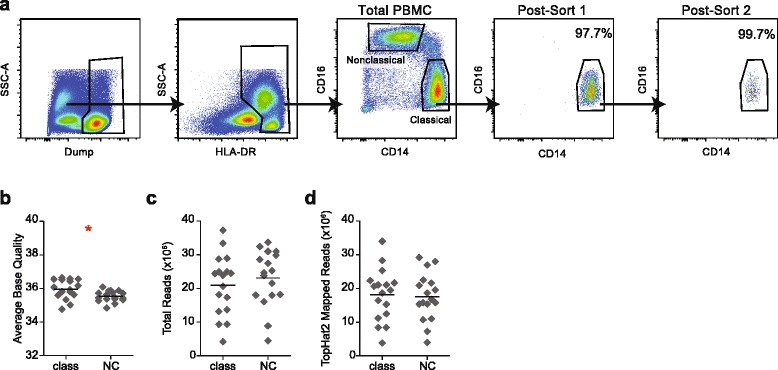
Monocyte isolation by flow cytometry and sequence read characteristics. **a** Gating strategy for isolation of monocyte subsets, and a representative demonstration of increasing purity of monocyte subsets upon successive rounds of flow cytometric sorting. **b** Average base quality across all bases within a sample. **c** Total reads per sample. **d** Representative example of total reads mapped to the human genome. Class, classical monocytes; NC, nonclassical monocytes

We sought to empirically assess performance characteristics of RNA-Seq analysis workflows applied to patient-derived clinical samples, which integrate multiple sources of variability that are not well represented in typical benchmarking datasets. We began by generating a test set of RNA-Seq profiles from purified human leukocytes. Specifically, we isolated cell populations from cryopreserved PBMCs collected as part of a study of malaria exposure in Ugandan children [26]. From these samples, we isolated CD177^−^CD7^−^CD19^−^HLA-DR^+^CD14^hi^CD16^−^ classical monocytes (also known as “inflammatory” monocytes) and CD177^−^CD7^−^CD19^−^HLA-DR^+^CD14^lo^CD16^+^ nonclassical monocytes (also known as “patrolling” monocytes) to high purity using two successive rounds of flow cytometry, which achieves >99% purity (Fig. 1a). Total RNA was isolated and processed into RNA-Seq libraries using SMARTer cDNA synthesis and Nextera fragmentation and indexing. Individual samples were multiplexed and sequenced as 51 bp single-end reads on an Illumina HiSeq 2500. Average base quality was relatively consistent across all samples, and although there was a statistically significant difference in average base quality between the classical and nonclassical monocyte groups, the effect size was small, with an absolute quality score difference of 0.4 between means (Fig. 1b). Total reads were variable, ranging from 4 to 37 million reads per sample, but with no significant difference between the classical and nonclassical groups (Fig. 1c). The absolute number of reads mapped by the read aligners likewise exhibited a wide range within each group, but without a significant difference between the groups (Fig. 1d).

### Overview of empirical testing

**Fig. 2:**
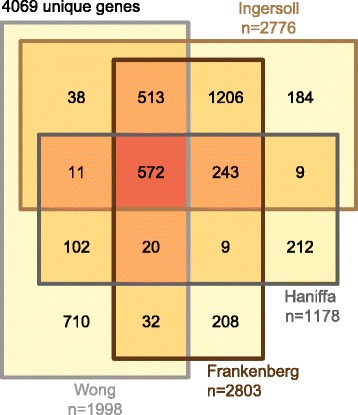
Concordance between significant gene expression differences between classical and nonclassical monocytes identified in four independent studies. Venn diagram showing degree of overlap of genes identified as significant by both SAM and limma from each microarray or BeadChip study

Several studies have previously explored gene expression differences between CD14^hi^CD16^−^ classical monocytes and CD14^lo^CD16^+^ nonclassical monocytes using microarray or BeadChip analysis [22–25]. Similar to our RNA-Seq dataset, these studies all represent monocytes from healthy donors. However, given that the data originate from labs in Singapore, the United States, and Germany, it is likely that there is some bias in genetics across the studies. It is also likely that these microarray data do not reflect the same genetic makeup and environmental pressures present in our data, which are obtained from Ugandan children with a high degree of malaria exposure. It should also be noted that recent studies have differentiated between three, rather than two, monocyte subsets [61], and several reference datasets were produced prior to this advancement and thus might not represent the same degree of purity in their nonclassical monocyte subset [22, 24, 25]. Despite these differences, in aggregate, these datasets provide a strong reference of biological ‘truth’ for comparison, as individual datasets can be evaluated as independent assessments of a given RNA-Seq analysis workflow. Because differentially expressed gene lists were not available for all studies and statistical criteria differed between studies, we have made our re-analysis of these publicly available datasets available as supplementary data (Additional file 2). Overall, the four datasets identified 4069 unique genes. Of these, 572 were shared among all 4 datasets, and 2755 were shared between at least two datasets. The Wong dataset showed the least overlap with the other datasets, contributing approximately half of the genes unique to a single dataset (Fig. 2).

With these four datasets as our references for performance comparisons, we focused our evaluation on RNA-Seq analysis approaches that have gained wide adoption due to their performance, availability, documentation, and/or ease of implementation. We evaluated 9 read aligners, 12 expression modelers and 13 methods for identifying differentially expressed genes and transcripts (Table 1), in all possible combinations. Exceptions included cases in which the output of an earlier stage was incompatible as the input to a later stage due to file format or expression units, or difficulty with software execution. In total, including comparisons made at the gene level and transcript level, and comparisons using expression data reported in counts, TPMs, or FPKMs, we evaluated 495 unique workflows (Additional File 4). We note that some of the workflows were not intended to be used in the resulting combinations by the original authors of the software.

Despite the aforementioned heterogeneity in the microarray and BeadChip analysis results, we found that performance of various RNA-Seq workflows was remarkably consistent across all four reference datasets. We note, however, that these reference datasets are also subject to the inherent biases of the experimental and computational methods used to produce them. Here, we have depicted our results using performance metrics averaged across all four references; however, we have also made available the performance estimates for each individual reference (Additional file 5 and Additional file 6), and an interactive visualization to explore the relative performance of the tools in more detail (Additional file 7).

### Differential influence of workflow stages

**Fig. 3:**
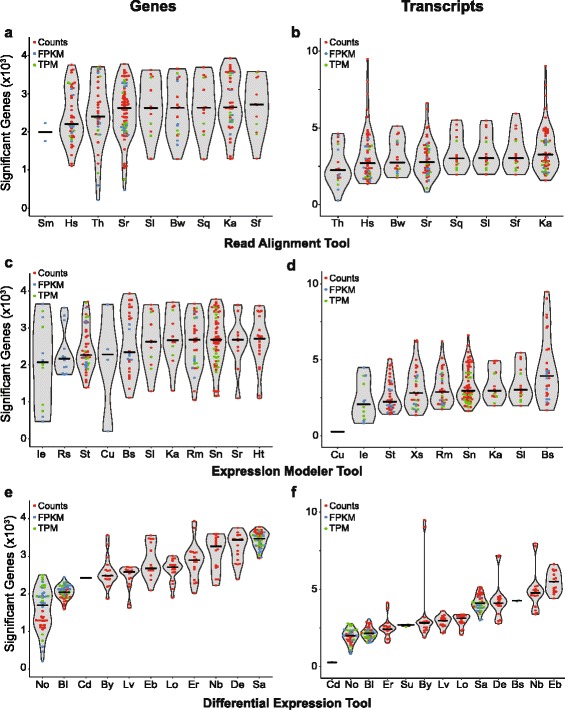
Number of significant genes predicted by workflows using a given method. The number of genes predicted by each workflow using a given read aligner (**a**, **b**), expression modeler (**c**, **d**), or differential expression tool (**e**, **f**), split by analyses run at the gene (**a**, **c**, **e**) or transcript (**b**, **d**, **f**) level. Each point represents a single workflow; line shows median

For each workflow consisting of all three steps (read alignment, expression modeling, and identification of differentially expressed genes), we evaluated the ability to detect genes differentially expressed between classical and nonclassical monocytes. When workflows identified a differentially expressed transcript, the corresponding gene was annotated as significant for performance evaluations, regardless of the status of other transcripts of the gene. In general, more significant genes were observed when evaluations were performed at the transcript level, because there are more transcripts than genes to potentially be differentially expressed. We have separated the analyses performed at the gene and transcript levels to highlight this difference throughout, and recommend that direct comparisons across these units not be made. Across workflows, we observed substantial variability in the number of differentially expressed genes identified (*n* = 208 to 9,489 significant genes; Fig. 3 and Additional file 5). Beyond the overall variation, two trends were apparent when the number of genes identified was examined on a by-tool basis. First, the differential expression tool had a larger impact on the number of genes identified than the read aligner and expression modeler (Fig. 3), as demonstrated by the relative homogeneity of range, distribution, and medians of the first two steps compared to the more variable parameters for the final step. Consequently, the coefficient of variation of the medians was largest for differential expression tools, as compared to read aligners and expression modelers, when assessed at both the gene level (20.5 versus 9.9 and 9.8, respectively) and the transcript level (43.4 versus 10.8 and 39.3). Second, differential expression tools varied in their robustness to different inputs, with some tools exhibiting relatively reproducible predictions regardless of the read aligner and expression modeler choices and expression units (e.g., Ballgown), and other differential expression analysis tools exhibiting a wide range of predictions as the input parameters varied (e.g., NOISeqBIO at the gene level) (Fig. 3e, f).

**Fig. 4:**
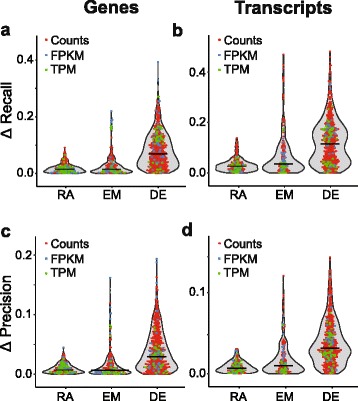
Impact of individual stages of the workflow on overall performance characteristics. The difference in recall (**a, b**) and precision (**c**, **d**) was calculated for exhaustive pairwise comparisons of workflows in which the software used for the given stage under evaluation was varied while the two other tools were held constant. The points reflect each absolute difference; the line represents the median. RA, read aligner; EM, expression modeler; DE, differential expression

We also evaluated performance of the workflows by calculating recall (intersecting significant genes divided by total number of significant reference genes) and precision (intersecting significant genes divided by total number of significant genes identified by RNA-Seq), using the microarray datasets as references. In order to further examine the influence of each stage of the workflow on the prediction of differentially expressed genes, we computed the absolute difference in recall and precision in all possible pairwise comparisons of workflows differing in only one component. Similar to the impact on the number of genes identified, for both precision and recall, the largest effects were observed in workflows differing in the statistical analysis of differential expression, as indicated by the increased medians of differences for this step (Fig. 4).

### Heterogeneity in performance characteristics of different workflows

**Fig. 5:**
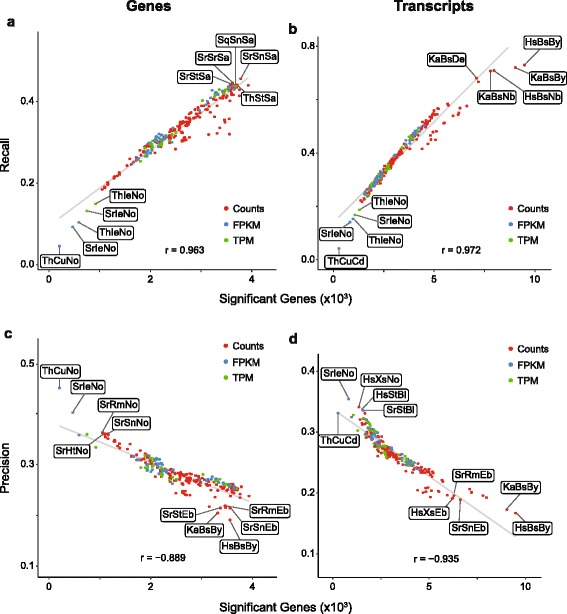
Relationship of recall and precision with number of genes predicted. Recall (**a**, **b**) and precision (**c**, **d**), plotted vs number of significant genes predicted by each workflow. Pearson *r* values are shown

We next evaluated performance by examining the specific recall and precision for individual workflows. Recall across the workflows was highly correlated with the number of genes identified (Fig. 5a, b). This was true regardless of which of the reference datasets was used for comparison (Additional file 5 and Additional file 6). Furthermore, the relative rankings of the workflows, ordered by absolute recall value, tended to be consistent across reference datasets (Additional file 6). For gene-level predictions, a subset of workflows using SAMseq exhibited the highest recall values; for transcript-level predictions, workflows using baySeq and NBPSeq exhibited the highest recall (Fig. 5a, b). However, there were exceptions to these rules, depending on the choice of read aligner and expression modeler (Fig. 5 and Additional file 6).

Precision was highly inversely correlated with the number of genes predicted across the workflows (Fig. 5c, d). Like recall, rankings were generally consistent regardless of which reference dataset was used, as was the overall relationship between significant genes and precision (Additional file 5 and Additional file 6). For gene-level predictions, a subset of workflows using NOISeqBIO exhibited the highest precision, whereas for transcript-level predictions those with the highest precision used several different combinations of tools, with the most prevalent being Ballgown and NOISeqBIO. Strikingly, when used on transcript-level data, the commonly used combination of TopHat2, cufflinks and cuffdiff exhibited one of the highest precision values, coupled with the second lowest number of differentially expressed genes identified (Fig. 5 and Additional file 5).

### Performance tradeoff

**Fig. 6:**
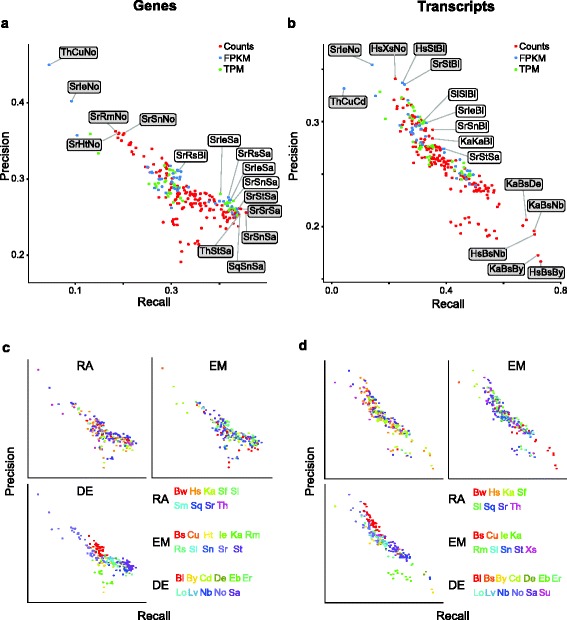
Comparison of performance metrics. **a**, **b** Precision and recall for each workflow, with top (*shaded*) and balanced (*white*) performers labeled. **c**, **d** Plots as above, with points colored by tool for each step

It is important to note that the specific workflows highlighted above are at the extremes of one or another performance metric. As would be expected, the prediction of more or fewer significant genes results in a tradeoff between recall and precision. For example, the workflows employing NOISeqBIO that exhibit the highest precision were also among those with the lowest recall (Fig. 5 and Additional file 6). An investigation of the relationship between precision and recall revealed that this tradeoff generally persisted throughout, with many workflows following an inverse linear relationship between precision and recall (Fig. 6a, b). This held true for both gene- and transcript-level analysis, was true regardless of the expression estimation units, and was also consistent across reference datasets (Fig. 6a, b, Additional file 7, and Additional file 8).

As observed previously with the number of significant genes and performance differences by step, the differential expression step had the greatest impact on the performance of each workflow along the spectrum of recall and precision (Fig. 6c, d). Specific tools that tended to track along this linear tradeoff were Ballgown, DESeq2, limma + voom, limma + vst and SAMseq; baySeq and EBseq consistently deviated the furthest. SAMseq, one tool with a nonparametric approach, has been highlighted as a high performer previously [3, 16], in particular when there are a large number of replicates available to approximate the underlying distribution, as is the case here; it performs well, though it does exhibit a tendency toward higher recall at the expense of precision. NOISeqBIO, the other tested differential expression tool that assumes a nonparametric distribution, has previously been observed to identify fewer differentially expressed genes with larger sample sizes [3]; we also observe this, as well as correspondingly low recall values. Of the differential expression methods tested, baySeq and EBseq are the most similar to each other in underlying statistical methodology; both use an underlying negative binomial model, and then estimate a posterior probability of being differentially expressed for each gene [46, 48]. The observation that EBseq deviated furthest from the precision/recall performance line, due to decreased precision without gains in recall, is similar to previous observations showing that EBSeq tended to produce many false positives with large sample sizes [16]. When applied to gene-level data, baySeq performed similarly to EBseq though not as extreme, with relatively low recall without commensurate gains in precision, which may reflect the similarity in their underlying methods. The development of Ballgown drew on the limma statistical methodologies based on linear models, although only Ballgown (and not limma) can accept TPM and FPKM data, in addition to counts. All three linear model workflows perform well and track along the linear precision/recall tradeoff, irrespective of upstream processing. However, there is some difference in default tuning, as Ballgown results tended towards higher precision, whereas limma + voom and limma + vst tended towards higher recall.

Aligners and estimators generally did not follow any specific trends, consistent with our observation that their influence is overshadowed by that of the differential expression analysis tool. However, two exceptions stood out. First, using BitSeq as the expression modeler tended to result in identification of large numbers of differentially expressed genes, but only in combination with differential expression tools that used an underlying negative binomial model for expression data (BaySeq, DESeq2, edgeR, and NBPSeq); EBSeq was the one exception, with the number of differentially expressed genes within range of workflows using differential expression tools that model other distributions (Ballgown, BitSeq, limma, and NOISeqBIO). We note that BitSeq was unusual in that its most prevalent estimated expression count value was between 1 and 2, rather than less than 1 as most expression modelers estimated; this likely explains why these expression data were poorly modeled by a negative binomial distribution. Second, using STAR as the read aligner, most notably with Ballgown as the differential expression tool, led to some of the highest performance workflows having a balance of precision and recall. Interestingly, these best performing workflows are not combinations of aligner and estimator that are suggested by the Ballgown authors, demonstrating the utility of broad, empirical exploration for uncovering improved workflows. Overall, there are multiple workflows that exhibit excellent performance, and, the relationship between recall and precision among the differential expression workflows that track along the inverse linear relationship likely reflects differential calibration of these methods with regard to the tradeoff between sensitivity and specificity, rather than any fundamental difference in statistical or algorithmic performance.

The above observations also suggest that the selection of a specific workflow should be largely influenced by the tolerance of a specific application for type I versus type II errors. However, it is also important to note that a significant number of workflows deviated from the roughly linear relationship between recall and precision, particularly for tools targeted at gene-level analyses; such workflows could be considered to exhibit lower performance, as higher performance workflows would be available as alternatives at a given recall or precision target value. Furthermore, our findings reflect a defined set of parameters, such as read length, sequencing coverage, sample number, and genetic polymorphism. Thus, it is possible that the performance, both absolute and relative, of the above workflows could vary under other conditions, as some studies have observed [8, 16]; as such, additional studies comparing workflow performance will be required to understand the generalizability of our observations. Importantly, when selecting a pipeline it is essential to consider not only the specific tools selected at each stage of the workflow, but also how they interact with one another.

## Conclusions

The choice of RNA-Seq analysis workflow, applied to genotypically heterogeneous samples, exerts significant influence on the repertoire, recall, and precision of the differentially expressed gene set that is identified. The impact of software selection at each step was not simply a function of upstream position in the workflow; rather, the choice of differential expression analysis approach exhibited the strongest impact on recall and precision, with more modest influences from the read aligner and expression modeler. The ultimate choice of workflow should take into consideration how the results will be used, and the performance characteristics described in this study. These, used in conjunction with consideration of the tolerance of the downstream applications for type I and type II errors, can guide the selection of an appropriate workflow. The data generated in this study also provide a useful benchmarking set for further development of RNA-Seq analysis tools and workflows.

## Additional files

Additional file 1: Table of software tools, with versions and runtime parameters. (XLSX 16 kb)
Additional file 2: Table of genes differentially expressed between nonclassical and classical monocytes in four reference studies, identified using limma and SAM, and their intersections with each other and annotated features in GRCh37. (XLSX 2721 kb)
Additional file 3: Figure of similarity in performance characteristics of significant gene identification by limma and SAM. Ranks of absolute precision and recall are shown for each workflow, when comparing SAM and limma microarray analysis of the reference datasets (a), comparing SAM and the intersection of SAM and limma (b), or comparing limma and the intersection of SAM and limma (c). (PDF 785 kb)
Additional file 4: Figure of all workflow and unit combinations run. (a) Gene-level workflows. (b) Transcript-level workflows. (PDF 718 kb)
Additional file 5: Table of number of significant genes identified, for each workflow against each reference dataset. (XLSX 46 kb)
Additional file 6: Table of workflow performance, including values and ranks for recall and precision, for each workflow against each reference dataset. (XLSX 189 kb)
Additional file 7: Interactive figure of comparison of performance metrics. (a) Absolute precision and recall for each workflow. (b) Relative ranks of precision and recall for each workflow. (XLSX 688 kb)
Additional file 8: Figure of recall and precision, for each reference dataset. Precision and recall as assessed using the Ingersoll (a, b), Haniffa (c, d), Frankenberger (e, f), and Wong (g, h) references, with top (shaded) and balanced (white) performers labeled. (PDF 185 kb)

## Abbreviations

RNA-Seq: RNA sequencing
PBMCs: Peripheral blood mononuclear cells

## References

[1] Robertson G, Schein J, Chiu R, Corbett R, Field M, Jackman SD (2010). De novo assembly and analysis of RNA-seq data. Nat Methods.

[2] Grabherr MG, Haas BJ, Yassour M, Levin JZ, Thompson DA, Amit I (2011). Full-length transcriptome assembly from RNA-Seq data without a reference genome. Nat Biotechnol.

[3] Seyednasrollah F, Laiho A, Elo LL (2015). Comparison of software packages for detecting differential expression in RNA-seq studies. Brief Bioinform.

[4] Pepke S, Wold B, Mortazavi A (2009). Computation for ChIP-seq and RNA-seq studies. Nat Methods.

[5] Oshlack A, Robinson MD, Young MD (2010). From RNA-seq reads to differential expression results. Genome Biol.

[6] Poplawski A, Marini F, Hess M, Zeller T, Mazur J, Binder H (2016). Systematically evaluating interfaces for RNA-seq analysis from a life scientist perspective. Brief Bioinform..

[7] Garber M, Grabherr MG, Guttman M, Trapnell C (2011). Computational methods for transcriptome annotation and quantification using RNA-seq. Nat Methods.

[8] Kanitz A, Gypas F, Gruber AJ, Gruber AR, Martin G, Zavolan M (2015). Comparative assessment of methods for the computational inference of transcript isoform abundance from RNA-seq data. Genome Biol.

[9] Fonseca NA, Marioni J, Brazma A (2014). RNA-Seq gene profiling—a systematic empirical comparison. PLoS One.

[10] Engström PG, Steijger T, Sipos B, Grant GR, Kahles A, Rätsch G (2013). Systematic evaluation of spliced alignment programs for RNA-seq data. Nat Methods.

[11] Palmieri N, Nolte V, Suvorov A, Kosiol C, Schlötterer C (2012). Evaluation of different reference based annotation strategies using RNA-Seq — a case study in drososphila pseudoobscura. PLoS One.

[12] Benjamin AM, Nichols M, Burke TW, Ginsburg GS, Lucas JE (2014). Comparing reference-based RNA-Seq mapping methods for non-human primate data. BMC Genomics.

[13] Reddy R. A Comparison of Methods: Normalizing High-Throughput RNA Sequencing Data. bioRxiv. 2015;026062.

[14] Kvam VM, Liu P, Si Y (2012). A comparison of statistical methods for detecting differentially expressed genes from RNA-seq data. Am J Bot.

[15] Zhang ZH, Jhaveri DJ, Marshall VM, Bauer DC, Edson J, Narayanan RK (2014). A comparative study of techniques for differential expression analysis on RNA-Seq data. PLoS One.

[16] Soneson C, Delorenzi M (2013). A comparison of methods for differential expression analysis of RNA-seq data. BMC Bioinformatics.

[17] Tang M, Sun J, Shimizu K, Kadota K (2015). Evaluation of methods for differential expression analysis on multi-group RNA-seq count data. BMC Bioinformatics.

[18] Yang C, Wu P-Y, Tong L, Phan JH, Wang MD (2015). The impact of RNA-seq aligners on gene expression estimation. ACM BCB.

[19] Nookaew I, Papini M, Pornputtapong N, Scalcinati G, Fagerberg L, Uhlén M (2012). A comprehensive comparison of RNA-Seq-based transcriptome analysis from reads to differential gene expression and cross-comparison with microarrays: a case study in Saccharomyces cerevisiae. Nucleic Acids Res.

[20] Teng M, Love MI, Davis CA, Djebali S, Dobin A, Graveley BR (2016). A benchmark for RNA-seq quantification pipelines. Genome Biol.

[21] Robert C, Watson M (2015). Errors in RNA-Seq quantification affect genes of relevance to human disease. Genome Biol.

[22] Ingersoll MA, Spanbroek R, Lottaz C, Gautier EL, Frankenberger M, Hoffmann R (2010). Comparison of gene expression profiles between human and mouse monocyte subsets. Blood.

[23] Wong KL, Tai JJ-Y, Wong W-C, Han H, Sem X, Yeap W-H (2011). Gene expression profiling reveals the defining features of the classical, intermediate, and nonclassical human monocyte subsets. Blood.

[24] Haniffa M, Shin A, Bigley V, McGovern N, Teo P, See P (2012). Human tissues contain CD141hi cross-presenting dendritic cells with functional homology to mouse CD103+ nonlymphoid dendritic cells. Immunity.

[25] Frankenberger M, Hofer TPJ, Marei A, Dayyani F, Schewe S, Strasser C (2012). Transcript profiling of CD16-positive monocytes reveals a unique molecular fingerprint. Eur J Immunol.

[26] Kamya MR, Arinaitwe E, Wanzira H, Katureebe A, Barusya C, Kigozi SP (2015). Malaria transmission, infection, and disease at three sites with varied transmission intensity in Uganda: implications for malaria control. Am J Trop Med Hyg.

[27] Matz M, Shagin D, Bogdanova E, Britanova O, Lukyanov S, Diatchenko L (1999). Amplification of cDNA ends based on template-switching effect and step-out PCR. Nucleic Acids Res.

[28] Petalidis L, Bhattacharyya S, Morris GA, Collins VP, Freeman TC, Lyons PA (2003). Global amplification of mRNA by template-switching PCR: linearity and application to microarray analysis. Nucleic Acids Res.

[29] Babraham Bioinformatics. FastQC at Babraham Bioinformatics [Internet]. Babraham Bioinforma. Available from: http://www.bioinformatics.babraham.ac.uk/projects/fastqc/. Accessed 7 May 2015.

[30] Langmead B, Salzberg SL (2012). Fast gapped-read alignment with bowtie 2. Nat Methods.

[31] Kim D, Langmead B, Salzberg SL (2015). HISAT: a fast spliced aligner with low memory requirements. Nat Methods.

[32] Bray NL, Pimentel H, Melsted P, Pachter L (2016). Near-optimal probabilistic RNA-seq quantification. Nat Biotechnol.

[33] Pertea M, Kim D, Pertea GM, Leek JT, Salzberg SL (2016). Transcript-level expression analysis of RNA-seq experiments with HISAT, StringTie and ballgown. Nat Protoc.

[34] Patro R, Mount SM, Kingsford C (2014). Sailfish enables alignment-free isoform quantification from RNA-seq reads using lightweight algorithms. Nat Biotechnol.

[35] Dobin A, Davis CA, Schlesinger F, Drenkow J, Zaleski C, Jha S (2013). STAR: ultrafast universal RNA-seq aligner. Bioinformatics.

[36] Kim D, Pertea G, Trapnell C, Pimentel H, Kelley R, Salzberg SL (2013). TopHat2: accurate alignment of transcriptomes in the presence of insertions, deletions and gene fusions. Genome Biol.

[37] Patro R, Duggal G, Love MI, Irizarry RA, Kingsford C. Salmon provides accurate, fast, and bias-aware transcript expression estimates using dual-phase inference. bioRxiv. 2016;021592.

[38] Jiang H, Wong WH (2008). SeqMap: mapping massive amount of oligonucleotides to the genome. Bioinformatics.

[39] Trapnell C, Hendrickson DG, Sauvageau M, Goff L, Rinn JL, Pachter L (2013). Differential analysis of gene regulation at transcript resolution with RNA-seq. Nat Biotechnol.

[40] Glaus P, Honkela A, Rattray M (2012). Identifying differentially expressed transcripts from RNA-seq data with biological variation. Bioinforma Oxf Engl.

[41] Anders S, Pyl PT, Huber W (2015). HTSeq—a python framework to work with high-throughput sequencing data. Bioinformatics.

[42] Nicolae M, Mangul S, Măndoiu II, Zelikovsky A (2011). Estimation of alternative splicing isoform frequencies from RNA-Seq data. Algorithms Mol Biol.

[43] Li B, Dewey CN (2011). RSEM: accurate transcript quantification from RNA-Seq data with or without a reference genome. BMC Bioinformatics.

[44] Jiang H, Wong WH (2009). Statistical inferences for isoform expression in RNA-Seq. Bioinforma Oxf Engl.

[45] Roberts A, Pachter L (2013). Streaming fragment assignment for real-time analysis of sequencing experiments. Nat Methods.

[46] Hardcastle TJ, Kelly KA (2010). baySeq: empirical Bayesian methods for identifying differential expression in sequence count data. BMC Bioinformatics.

[47] Love MI, Huber W, Anders S (2014). Moderated estimation of fold change and dispersion for RNA-seq data with DESeq2. Genome Biol.

[48] Leng N, Dawson JA, Thomson JA, Ruotti V, Rissman AI, Smits BMG (2013). EBSeq: an empirical Bayes hierarchical model for inference in RNA-seq experiments. Bioinformatics.

[49] Robinson MD, McCarthy DJ, Smyth GK (2010). edgeR: a Bioconductor package for differential expression analysis of digital gene expression data. Bioinformatics.

[50] Smyth GK (2004). Linear models and empirical bayes methods for assessing differential expression in microarray experiments. Stat Appl Genet Mol Biol..

[51] Di Y, Schafer DW, Cumbie JS, Chang JH (2011). The NBP negative binomial model for assessing differential gene expression from RNA-Seq. Stat Appl Genet Mol Biol..

[52] Tarazona S, Furió-Tarí P, Turrà D, Pietro AD, Nueda MJ, Ferrer A (2015). Data quality aware analysis of differential expression in RNA-seq with NOISeq R/Bioc package. Nucleic Acids Res.

[53] Li J, Tibshirani R (2013). Finding consistent patterns: a nonparametric approach for identifying differential expression in RNA-Seq data. Stat Methods Med Res.

[54] Pimentel HJ, Bray N, Puente S, Melsted P, Pachter L. Differential analysis of RNA-Seq incorporating quantification uncertainty. bioRxiv. 2016;058164. 10.1038/nmeth.4324 28581496

[55] wasabi [Internet]. GitHub. [cited 2016 Aug 31]. Available from: https://github.com/COMBINE-lab/wasabi.

[56] Soneson C, Love MI, Robinson MD (2015). Differential analyses for RNA-seq: transcript-level estimates improve gene-level inferences. F1000Research.

[57] Ancuta P, Liu K-Y, Misra V, Wacleche VS, Gosselin A, Zhou X (2009). Transcriptional profiling reveals developmental relationship and distinct biological functions of CD16+ and CD16- monocyte subsets. BMC Genomics.

[58] Tusher VG, Tibshirani R, Chu G (2001). Significance analysis of microarrays applied to the ionizing radiation response. Proc Natl Acad Sci U S A.

[59] Kim CC, Falkow S (2003). Significance analysis of lexical bias in microarray data. BMC Bioinformatics.

[60] Smyth GK, Gentleman R, Carey VJ, Huber W, Irizarry RA, Dudoit S (2005). Limma: linear models for microarray data. Bioinforma. Comput. Biol. Solut. Using R bioconductor [internet].

[61] Wong KL, Yeap WH, Tai JJY, Ong SM, Dang TM, Wong SC (2012). The three human monocyte subsets: implications for health and disease. Immunol Res.

