# Supplementary figures and images for: Empirical assessment of analysis workflows for differential expression analysis of human samples using RNA-Seq
Source: BMC Bioinformatics. 2017 Jan 17;18:38. doi: 10.1186/s12859-016-1457-z (PMC5240434; doi:10.1186/s12859-016-1457-z)

Precision

Recall

Genes

Transcripts

Genes

Transcripts

a

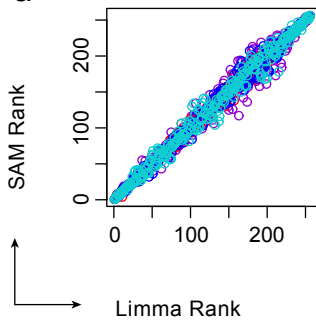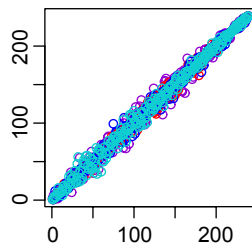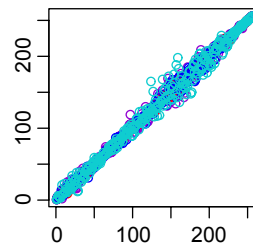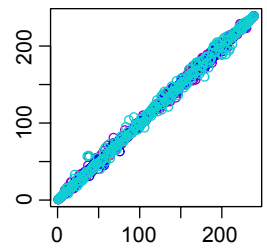

b

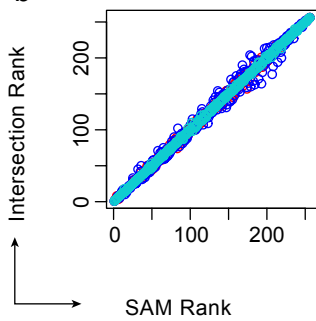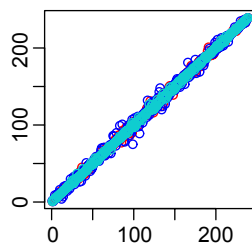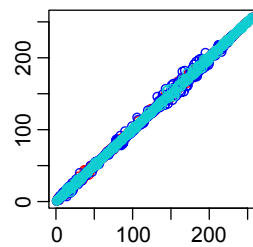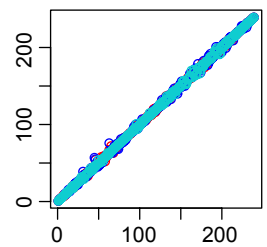

c

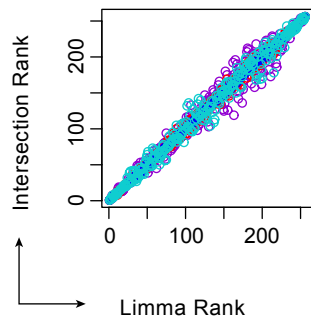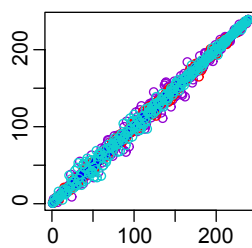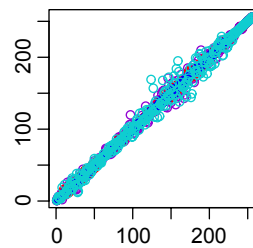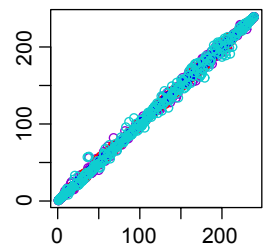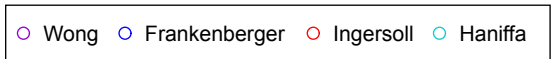

Supplement: Additional file 3: — Figure of similarity in performance characteristics of significant gene identification by limma and SAM. Ranks of absolute precision and recall are shown for each workflow, when comparing SAM and limma microarray analysis of the reference datasets (a), comparing SAM and the intersection of SAM and limma (b), or comparing limma and the intersection of SAM and limma (c). (PDF 785 kb) [file 12859_2016_1457_MOESM3_ESM.pdf]

Ingersoll

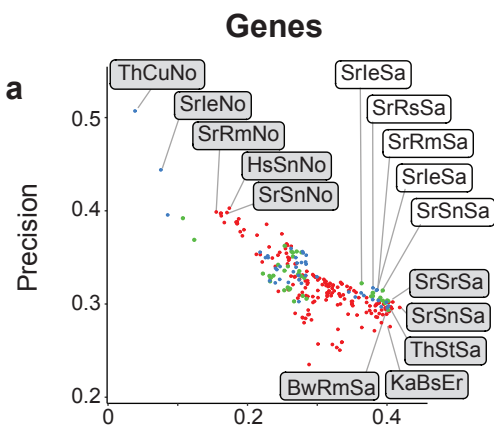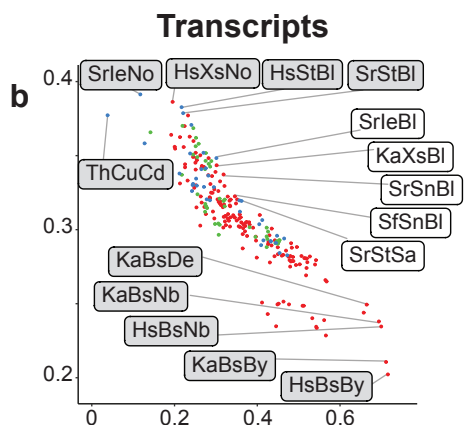

Haniffa

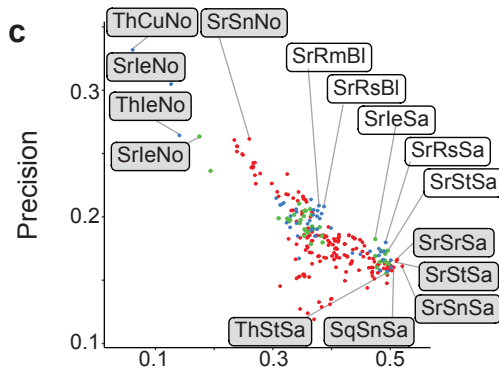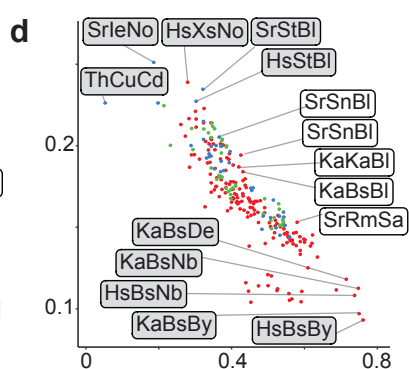

Frankenberger

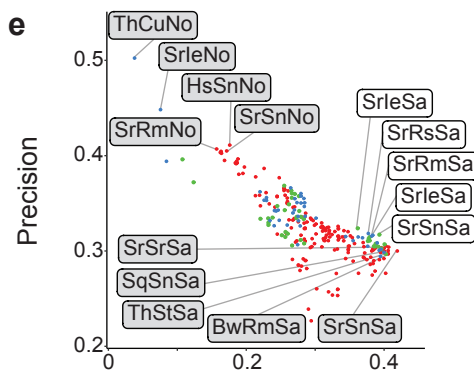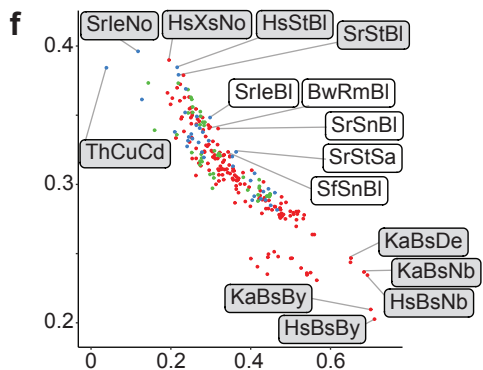

Wong

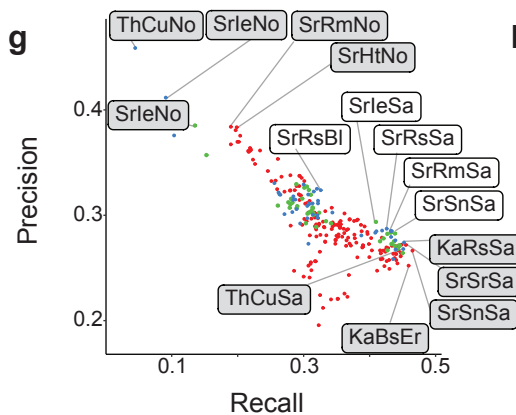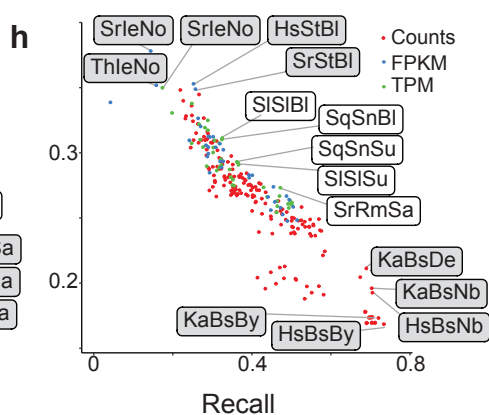

Supplement: Additional file 8: — Figure of recall and precision, for each reference dataset. Precision and recall as assessed using the Ingersoll (a, b), Haniffa (c, d), Frankenberger (e, f), and Wong (g, h) references, with top (shaded) and balanced (white) performers labeled. (PDF 185 kb) [file 12859_2016_1457_MOESM8_ESM.pdf]
