# Supplementary material for: Empirical assessment of analysis workflows for differential expression analysis of human samples using RNA-Seq
Source: BMC Bioinformatics. 2017 Jan 17;18:38. doi: 10.1186/s12859-016-1457-z (PMC5240434; doi:10.1186/s12859-016-1457-z)
Supplement: Additional file 4: — Figure of all workflow and unit combinations run. (a) Gene-level workflows. (b) Transcript-level workflows. (PDF 718 kb) [file 12859_2016_1457_MOESM4_ESM.pdf]

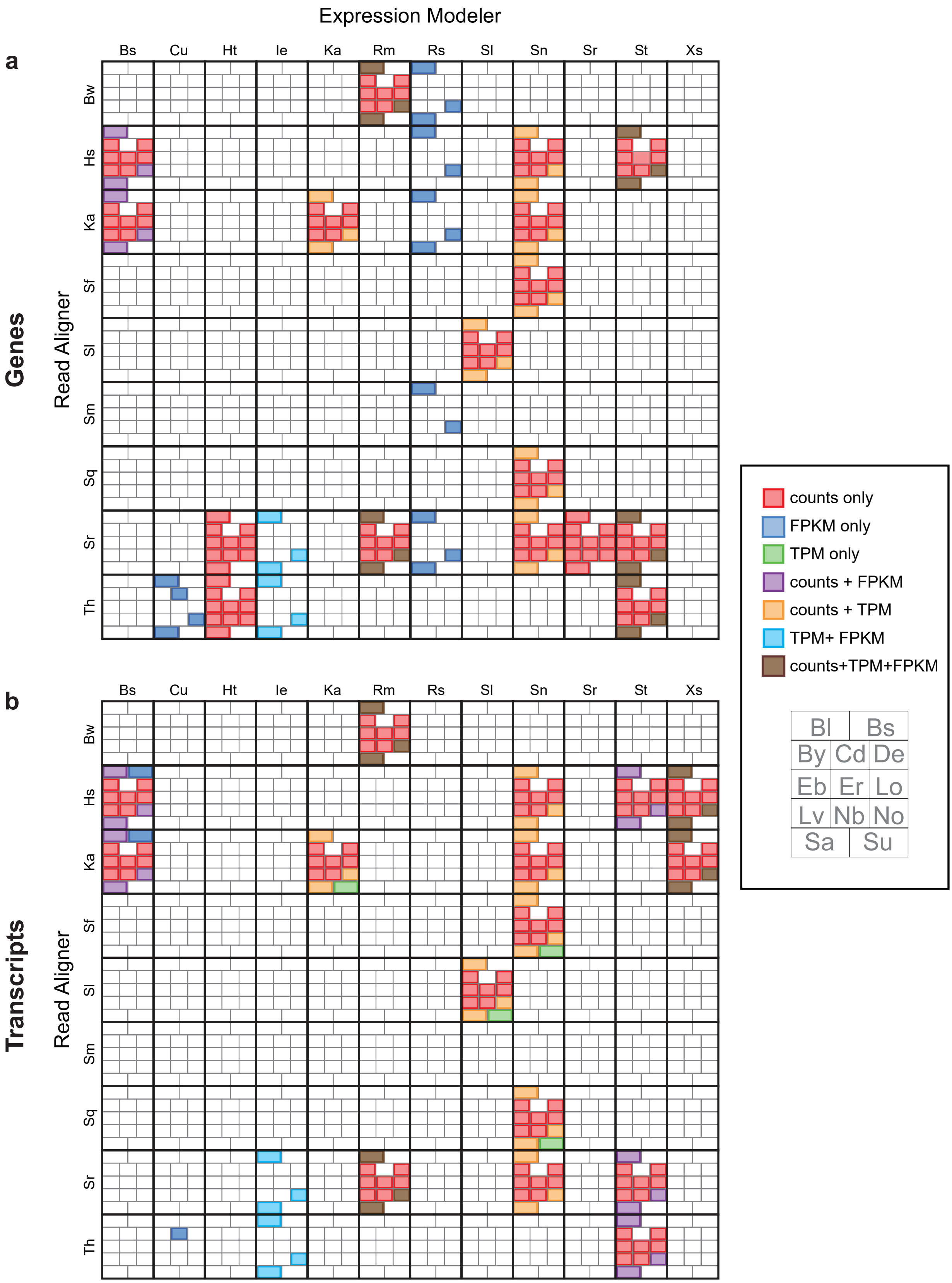
Expression Modeler
a
Genes
Read Aligner
Bs
Cu
Ht
Ie
Ka
Rm
Rs
Sl
Sn
Sr
St
Xs
Bw
Hs
Ka
Sf
Sl
Sm
Sq
Sr
Th
b
Transcripts
counts only
FPKM only
TPM only
counts + FPKM
counts + TPM
TPM+ FPKM
counts+TPM+FPKM
Bl
Bs
By
Cd
De
Eb
Er
Lo
Lv
Nb
No
Sa
Su
